# CellMap visualizes protein-protein interactions and subcellular localization

**DOI:** 10.12688/f1000research.12707.2

**Published:** 2018-02-01

**Authors:** Christian Dallago, Tatyana Goldberg, Miguel Angel Andrade-Navarro, Gregorio Alanis-Lobato, Burkhard Rost

**Affiliations:** 1 Department of Informatics, Bioinformatics & Computational Biology , TUM (Technical University of Munich), Munich, Germany; 2 Center for Doctoral Studies in Informatics and its Applications (CeDoSIA), TUM (Technical University of Munich) Graduate School, Munich, Germany; 3 Faculty of Biology, Johannes Gutenberg University Mainz, Mainz, Germany; 4 Department of Biochemistry and Molecular Biophysics, Columbia University, New York City, NY, USA; 5 Institute for Food and Plant Sciences WZW, Freising, Germany; 6 Institute for Advanced Study (TUM-IAS), Munich, Germany

**Keywords:** subcellular location, biological visualization, protein-protein interaction

## Abstract

Many tools visualize protein-protein interaction (PPI) networks. The tool introduced here, CellMap, adds one crucial novelty by visualizing PPI networks in the context of subcellular localization, i.e. the location in the cell or cellular component in which a PPI happens. Users can upload images of cells and define areas of interest against which PPIs for selected proteins are displayed (by default on a cartoon of a cell). Annotations of localization are provided by the user or through our in-house database. The visualizer and server are written in JavaScript, making CellMap easy to customize and to extend by researchers and developers.

## Introduction

Many tools visualize different aspects of protein-protein interaction (PPI) networks; the most prominent might be Cytoscape ^1^. Existing visualizations of large PPI networks continue to be difficult to use. Some proteins interact with many hundreds or thousands of others. Often referred to as ‘PPI hairballs’, such hubs are in the way of understanding large data sets. Many ways have been proposed to resolve such hairballs through the addition of biologically meaningful dimensions such as pathways ^2^ or time ^3^.

Another dimension was first introduced a decade ago, namely the overlay of PPIs with subcellular localization ^4^. Combining PPI networks with protein location provide an intuitive way of laying out PPI networks on a graphical representation of the cell, and might reduce the clutter from PPI hairballs. This decade-old solution ^4^ no longer copes with today’s data, in terms of scalability nor of customizability and in terms of ease-of-use.

CellMap, the prototype introduced here, takes up on the idea of PPI visualization constrained by protein location, and provides a simple visual interface for users to explore protein location inside a cell. It presents this information in a graphically pleasant way and offers several customization features. The framework has been optimized to simplify future developments, such as the addition of further data dimensions (e.g. inclusion of protein trafficking). An instance of the tool with localization data from a previous publication that includes protein localizations of the human proteome ^5^ and PPI data from the Human Integrated Protein-Protein Interaction rEference (HIPPIE) resource ^6^ is available at http://cell.dallago.us.

## Methods

### Implementation

The CellMap prototype is an integrated portal that exposes API calls to retrieve images (representing cells) and protein information, as well as a frontend to visualize protein location and PPI data. The portal is fully written in JavaScript, namely in the JavaScript interpreter node.js ( https://nodejs.org) for the backend and vanilla JavaScript for the frontend. The portal is deployed to the public through a Docker container. Docker is a technology that allows shipping of packaged services such as web applications to customers and users without the need to install dependencies other than the Docker engine (available through: https://www.docker.com). For the representation of cell images as maps, the Leaflet framework is used. Leaflet is a JavaScript-based tool used to represent maps ( http://leafletjs.com).

**Figure 1.**
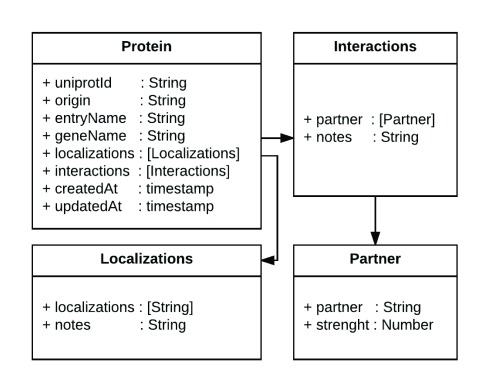
Diagram of the data representation in CellMap. In the figure we present a diagram of the Protein class, which contains several attributes of type *String*, two fields of type *timestamp* and ** two arrays (in square brackets) that reference the *Interactions* and *Localizations* classes. The arrows highlight the referenced models. This simple representation of information about a protein, its protein-protein interaction partners and its localizations enables the tool to be reused with one’s own datasets.

Data about proteins are stored as JSON documents in a Mongo ( http://mongodb.com) database. All information about the interaction partners and the subcellular localization of a protein is stored in a single JSON document, making the data structure simple to understand for non-experts and enabling them to deploy prototypes using their own data. Figure 1 schematically represents a protein data model (for a specific example for a protein object: http://cell.dallago.us/api/proteins/search/Q99943).

### Operation

**Figure 2.**
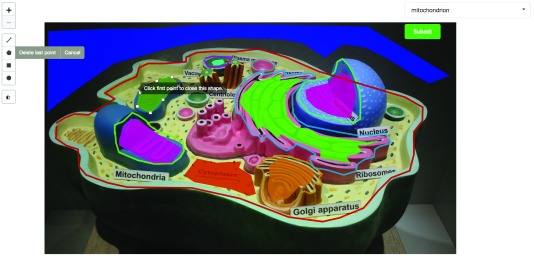
Section of a screenshot of the CellMap editing tool on a private instance of the portal. In the screenshot, an authorized user with editing capabilities draws a polygon (dark green) representing a new cellular compartment or region of interest (ROI). The user has a set of tools on the left side that can be used to draw polygons, lines, squares or circles. Once the new region has been drawn, the user can associate a cellular compartment through the dropdown input on the top-right and submit the new information to the server. The image used for this screenshot was taken from Wikimedia’s user Royroydeb, under CC BY-SA 4.0 ( http://bit.ly/2fuYRiE) and is used in this figure for demonstrative purposes only, as using it on the online version of CellMap would infringe copyright.

In CellMap, users can choose to upload new maps (images of cells). They can modify the location of regions of interest (ROIs) for a selected map ( Figure 2), and visualize the locations of selected proteins on a map or render protein-protein interaction networks from a set of selected proteins.

**Figure 3.**
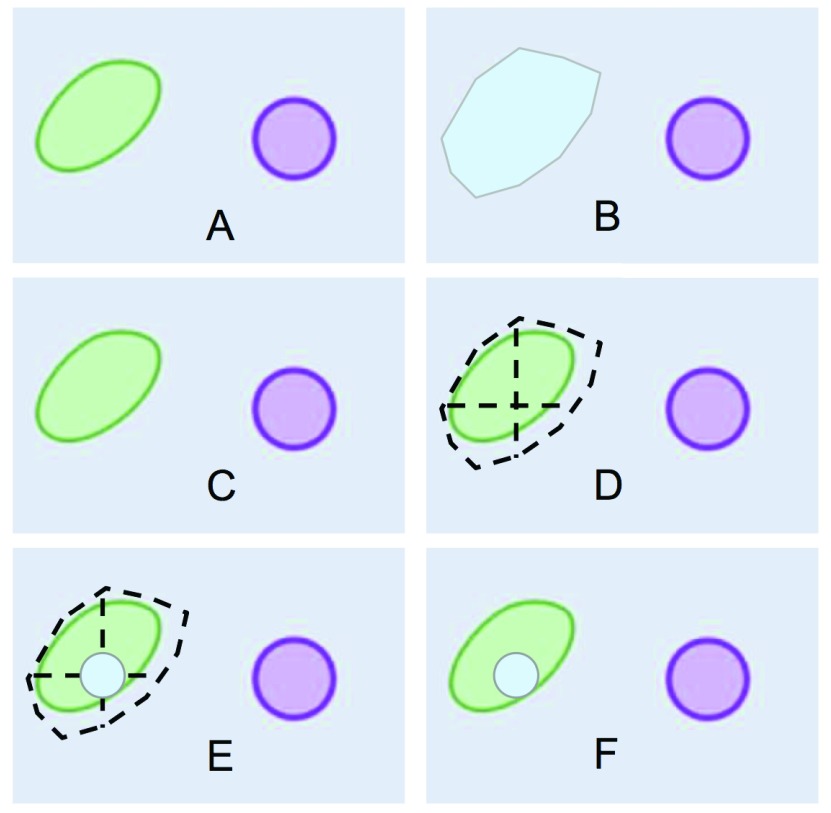
Definition of an area and drawing of protein circle. ( **A**) Section of a cartoon image of a cell; ( **B**) user-drawn polygon representing the area occupied by a vacuole; ( **C**) how the section of the cartoon image is displayed on the PPI/map viewer; ( **D**) random point calculation inside vacuole-polygon-defined area; ( **E**) drawing of a protein circle located inside the vacuole, ( **F**) result of loading a protein localized in the vacuole as shown by PPI/map viewer.

To maintain a consistent coloring scheme for different cellular compartments throughout a set of different images, each compartment is assigned a unique color through the hash of the compartment’s name (e.g. light blue = vacuole, Figure 3B). Using this coloring approach, users might eventually learn to associate color with compartment. When proteins are loaded into the map, they are assigned pseudo-random coordinates representing a point that lies within the boundaries of the ROI in which they are localized ( Figure 3D). A circle of a given radius is placed on the randomly generated point ( Figure 3E-F), and the circle will be filled with the same color as the compartment in which the protein is located in ( Figure 3B and 3F).

**Figure 4.**
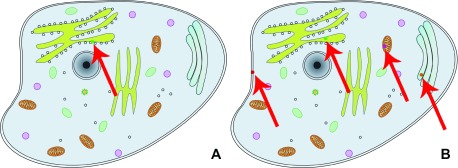
Comparison between PPI viewer and map viewer. The left view ( **A**) shows the PPI viewer, which depicts the result of loading protein Q9NR71 and displays a circle for the first localization found in the array of locations ( http://cell.dallago.us/ppi?p=Q9NR71); The right panel ( **B**) shows the Map viewer, which depicts the result of loading the same protein Q9NR71 and displays a circle for the protein in each of its reported location ( http://cell.dallago.us/map?p=Q9NR71). The red arrows are overlaid on top of the screenshots to highlight where the protein circles have been drawn in the viewers, since fitting the screenshot on the page reduces the overall size of the images.

**Figure 5.**
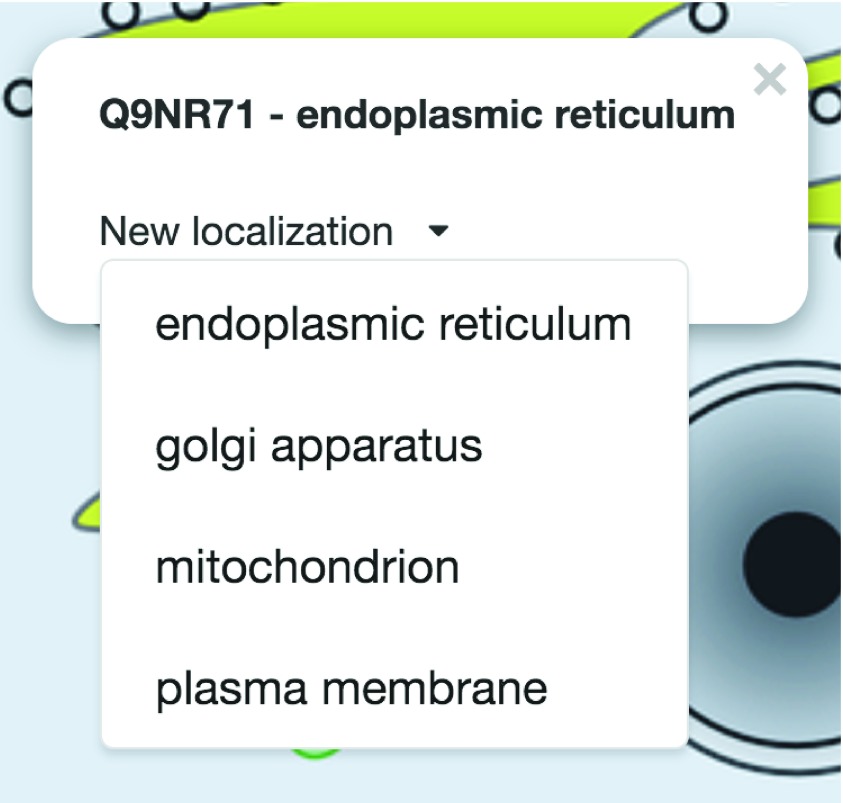
Protein information box. Top: information about the selected protein. Bottom: new localization selection box rendered in the PPI viewer when clicking on the protein circle ( http://cell.dallago.us/ppi?p=Q9NR71).

**Figure 6.**
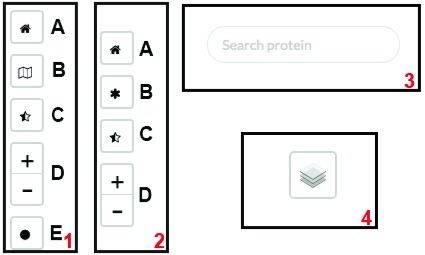
Controls used in the different viewers. ( **1**) Top-left controls of PPI viewer; ( **2**) top-left controls of map viewer; ( **3**) top-center search panel of PPI/map viewer; ( **4**) top-right layer control on PPI/map viewer.

Users can choose between two visualization options: the subcellular location in the context of the protein-protein interaction viewer (PPI viewer, Figure 4A, http://cell.dallago.us/ppi), and the protein subcellular location viewer (Map viewer, Figure 4B, http://cell.dallago.us/map). The two viewers can load the same images of cells (maps) and collect localization data from the same source, in the publicly available instance by 5. The PPI viewer offers the possibility to overlay networks between proteins being visualized. The map viewer displays all locations reported for a given protein simultaneously, while the PPI viewer only displays only one location at a time (by default: the first localization in the array of localizations as described in the protein data model, Figure 1); users can manually change the location by clicking on the protein circle and selecting a new location from the information box ( Figure 5). Both the PPI and the map viewer are enriched by several controls ( Figure 6): The top-left controls enable actions including: the navigation to the home of CellMap ( Figure 6, panels 1 and 2, A), switching from the map viewer to the PPI viewer and vice versa, keeping the proteins currently loaded in the view ( Figure 6, panels 1 and 2, B), reducing the opacity of the cell map, highlighting the protein circles ( Figure 6, panels 1 and 2, C), zooming in- and out of the map and PPI viewers ( Figure 6, panels 1 and 2, D), and visualization of the global network among all proteins loaded in the visualizer ( Figure 6, panel 1, E). The top-right control allows to temporarily hide loaded proteins or activate an overlay of the user-drawn localizations ( Figure 6, panel 4). The top-center search panel allows users to load new proteins by searching for their UniProt identifier, primary gene or primary protein name ^7^ into the viewer ( Figure 6, panel 3).

**Figure 7.**
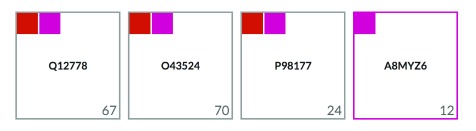
Results of searching for protein “ *foxo*”. The screenshot of this section of the home page shows four proteins that match the search criterion “ *foxo*” either by their UniProt identifier, primary gene name or primary protein name. The protein boxes contain the UniProt identifiers of the matched proteins (center) and display the number of interaction partners (bottom-right) and several color-filled boxes graphically representing the localizations reported for the matched proteins (top-left).

To facilitate the retrieval of proteins and their interacting partners, CellMap provides basic search functionalities. Users can search for proteins based on their UniProt identifiers, by their gene identifiers or by their protein names. When performing the search, the page renders a grid containing boxes, each representing a different protein ( Figure 7). Inside the boxes, the UniProt identifier for the protein that matched the search criterion is displayed. Starting on the top-right of every box a smaller colored square for each compartment is displayed in which that protein is localized. For proteins annotated to be in a single compartment, the border of the outer box (representing one protein as indicated by the UniProt ID in the center of the box) will get the color of that compartment (2 ^nd^ box in Figure 7). Clicking on one of the colored squares will filter results based on the compartment represented by that color. In the bottom-right of each box, the total number of PPI partners are annotated.

## Discussion

**Figure 8.**
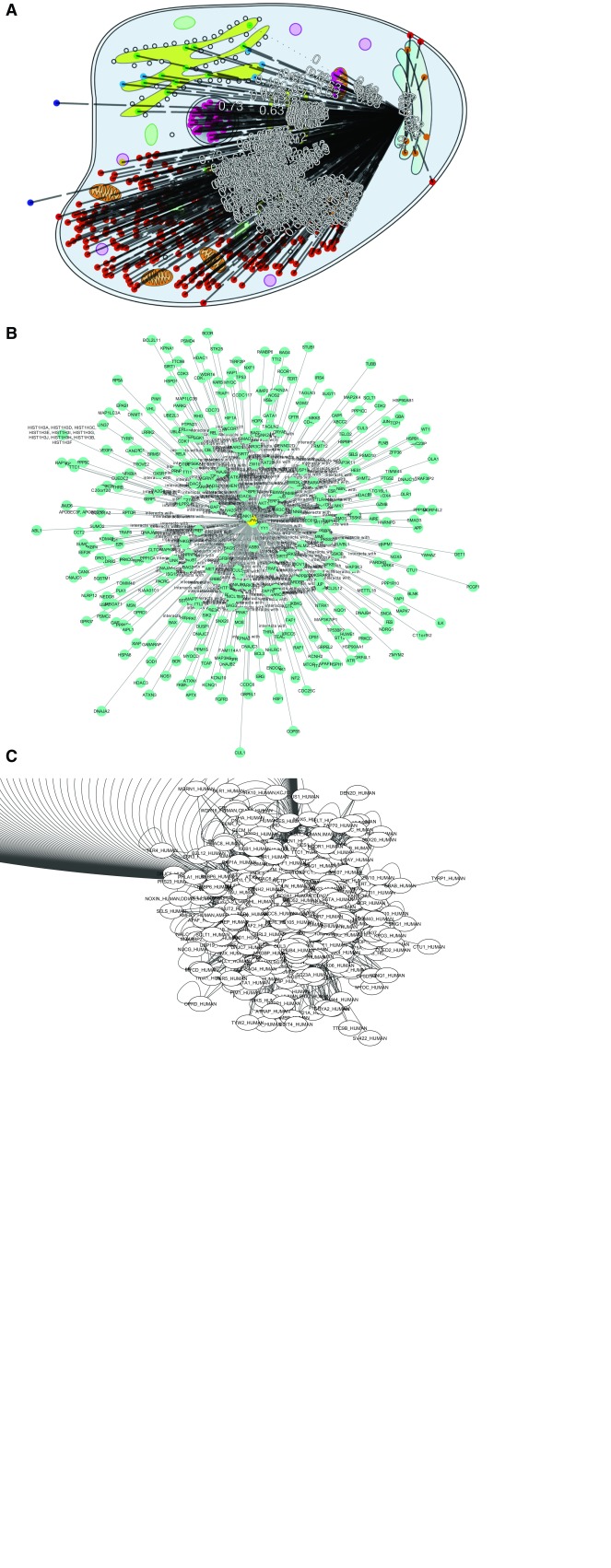
PPI hub in CellMap ( **A**), Cytoscape.js ( **B**) and Cytoscape desktop ( **C**). For HSPA4 (Heat shock 70 kDa protein 4, UniProt identifier P34932), we show some of the PPIs known (according to HIPPIE HSPA4 has 338 interaction partners). We chose this as one example of a protein with many more PPIs than the average protein (“PPI hub”). The figure compares how three different PPI viewers cope with the HSPA4 network: ( **A**) CellMap ( http://cell.dallago.us/protein/P34932), ( **B**) HIPPIE’s Cytoscape.js visualizer and ( **C**) the desktop version of Cytoscape. Proteins in CellMap are represented as colored dots on the map (image) of the cell, and upon selecting the protein of interest an overlay of edges is drawn. In Cytoscape and Cytoscape.js, proteins are represented as nodes containing a label (protein name as UniProt identifier), and edges are directly inferred from the data. The Cytoscape.js visualization was taken directly from HIPPIE. The Cytoscape network was automatically drawn upon loading the HIPPIE dataset and selecting the protein of interest and it’s direct neighbors.

Some CellMap functionality is exemplified by a heat shock protein (HSPA4; Heat shock 70 kDa protein 4, UniProt identifier P34932) with many interaction partners (338, according to HIPPIE, http://cbdm-01.zdv.uni-mainz.de/~mschaefer/hippie/query.php?s=HSPA4) in different compartments. The objective was to showcase how CellMap can simplify PPI hairballs. We visualize the same PPI network using CellMap ( Figure 8A) and Cytoscape ^1^ in the form of the Cytoscape.js version used by HIPPIE ( Figure 8B) and the Cytoscape desktop version ( Figure 8C).

None of the three viewers solves the PPI hairball problem completely. Without zooming in, the information density for 338 protein pairs is too high to be helpful. HIPPIE’s layout for Cytoscape.js ( Figure 8B) clearly improves over the standard Cytoscape desktop version ( Figure 8C) by centering the view around HSPA4, the protein of interest. In CellMap ( Figure 8A) the biologically relevant differences between pairs from the same and from different compartments remain visible.

By using a biologically relevant dimension (protein localization), instead of drawing nodes in positions based on edge weight (force layout of Cytoscape), some aspects of the protein and its partners become obvious at first glance, e.g. that HSPA4 interacts with many nuclear and cytoplasmic proteins, as well as with proteins that are secreted (extra-cellular) and located in the Endoplasmic Reticulum (ER, Figure 8). This may suggest the hypothesis HSPA4 to be an important hub involved in process spanning across compartments. Such a hypothesis is presented in our supplementary material (Figure SOM_1), where we analyze the visualization of the FOXO3 protein through CellMap.

One disadvantage of CellMap over the Cytoscape.js view is that the protein identifiers are not visible at all on the static image (protein identifiers become visible through mouse-over events in CellMap). However, in the image shown ( Figure 8) the Cytoscape.js names also remain unreadable. Another problem with CellMap are the numbers displayed on edges (experimental reliability of the PPI as given by HIPPIE). In our view, this information is extremely important to look at interactions, but we are still lacking a more sophisticated mechanism to visualize these numbers.

CellNetVis ^8^ is a recent tool that also connects localization with PPI networks. It emphasizes the way PPI networks are laid out through the adaptation of a so-called force-directed layout (using the tool While). Although CellMap and CellNetVis are founded on a similar idea, user experience and focus differ importantly. For instance, CellMap can be driven by data from users that define the number of compartments on a map, and provide localizations. In contrast, CellNetVis uses a fixed subset of compartments and an ad hoc diagram for the cell. Additionally, CellMap comes with out of the box data for the human proteome and allows the community to grow the tool by enriching datasets (images and localizations), whereas CellNetVis has a per-use approach, allowing to visualize networks stored in specialized XGMML files. Another unique aspect of CellMap is the openness to introduce further biologically meaningful dimensions (beyond location such as time or pathways) that increase the usefulness of PPI visualization tools to create new testable hypotheses.

## Conclusions

CellMap is a prototype providing a portal exploring the idea of using protein subcellular location as the basis to construct more complete visualizations of biological data, such as protein-protein interactions (PPIs). Using this paradigm, we claim that additional information, such as pathways, can be layered on top of the current visualization of subcellular location to potentially generate meaningful biological insights. The source code for the portal is publicly available and an instance of the portal with location data from a previous publication about the subcellular localization of the human proteome ^5^ and protein-protein interaction data from HIPPIE ^6^ ( http://cbdm-01.zdv.uni-mainz.de/~mschaefer/hippie) is running at http://cell.dallago.us. The visualization tool is written in JavaScript, thereby tapping into a very large user base for customized extensions and modifications. With the release of the prototype, we aim at creating a user base and awareness of the tool, ultimately collecting precious feedback from experimentalists and technical users alike.

## Abbreviations

2D: two dimensions, API: Application Program Interface, ID: identifier, JSON: JavaScript Object Notation, PPI: protein-protein interaction, ROI: region of interest.

## Software availability

The CellMap prototype is released as open source software under the GNU General Public License v3.0. Documentation, source code and viewer are available at https://github.com/sacdallago/cellmap. Archived source code as at the time of publication is available at https://doi.org/10.5281/zenodo.904324 ^9^. An example of use with protein localization data from a recent publication ^5^ and from the HIPPIE database of protein-protein interactions ^6^ is available at http://cell.dallago.us.
