# Supplementary material for: CellMap visualizes protein-protein interactions and subcellular localization
Source: F1000Res. 2018 Feb 1;6:1824. Originally published 2017 Oct 11. [Version 2] doi: 10.12688/f1000research.12707.2 (PMC5811672; doi:10.12688/f1000research.12707.2)
Supplement: Peer review history

# Peer review: Referee response for version 2

**DOI:** 10.5256/f1000research.15085.r30439

**Published:** 2018-02-12

**Authors:** Sandra Orchard

**Affiliations:** 1 European Bioinformatics Institute (EMBL-EBI), European Molecular Biology Laboratory, Cambridge, UK

The authors have addressed my concerns and I have no further comments to add.

I have read this submission. I believe that I have an appropriate level of expertise to confirm that it is of an acceptable scientific standard.

# Peer review: Referee response for version 1

**DOI:** 10.5256/f1000research.13762.r29754

**Published:** 2018-01-18

**Authors:** Augustin Luna

**Affiliations:** 1 Dana-Farber Cancer Institute, Harvard Medical School, Boston, MA, USA

The tool provides an interesting feature to help declutter visualizations of biological networks using localization information. Some comments:

- It would be good if the names of the used databases was stated in the last paragraph of the introduction.
- The tool would be more intuitive for new users, if it provided descriptions the various colors used on the site with the same explanation as in the paper. For example, the colored boxes that represent localizations in the search results and the dot colors used for the protein visualization on the cell map.
- It is unclear from the paper all the types of interactions might be shown in the represented networks.
- Also, it is unclear from the paper, what happens to the network visualization in the cases where the identified proteins are present in multiple locations.

I have read this submission. I believe that I have an appropriate level of expertise to confirm that it is of an acceptable scientific standard.

# Response

**Published:** 2018-01-29

**Authors:** Christian Dallago

**Affiliations:** Technical University of Munich, Germany

Dear Dr. Luna,

Thank you very much for your input on our work.

We have submitted a new version of the manuscript, which should address points one and four of your comment.

As to the second point: we have created a new feature item for our next release that displays a button on the map viewer to display a modal with the legend. As of now: a legend is available by scrolling down to the second half of the page in the map or ppi viewers (e.g. http://cell.dallago.us/map/573c87c182a9e1ae1e37d08e?p=P04637 ) and expanding the "Legend" tab. We understand that this can be overseen and improved, therefore we thank you for the input.

As to the third point: in this manuscript, we focus on discussing the software implementation and visualization abilities of CellMap, rather than the data sources used in the example deployment hosted on http://cell.dallago.us. More information about the types of interactions reported by the HIPPIE data source can be found in the latest paper describing HIPPIE ( http://nar.oxfordjournals.org/content/early/2016/10/28/nar.gkw985) and directly on the HIPPIE information page ( http://cbdm-01.zdv.uni-mainz.de/~mschaefer/hippie/information.php#sources).

Please, feel free to suggest any other changes to both our manuscript and tool.

Best regards,

Christian Dallago.

# Peer review: Referee response for version 1

**DOI:** 10.5256/f1000research.13762.r27544

**Published:** 2017-11-13

**Authors:** Sandra Orchard

**Affiliations:** 1 European Bioinformatics Institute (EMBL-EBI), European Molecular Biology Laboratory, Cambridge, UK

The authors describe a tool for visualising PPI networks in the context of the subcellular localisation of the searched protein.

1. I have twice tried to review this paper and both times the http://cell.dallago.us link gave me a MongoDB error. I have therefore had to review the paper without being able to view the tool. This is not satisfactory. I was unable to test the conclusions about the tool and its findings.
2. The tool uses a static interaction compilation database (HIPPIE) as the source of PPIs. Did the authors not consider using the PSICQUIC web service, which gives the users considerably more options as to where to source their PPI data from, and also allows the visualisation of protein-small molecule interactions and also potentially the site of action of protein-drug interactions, also available via PSICQUIC. It would also allow the data to be as up to date as the latest release of each database, which will be more frequent than releases of HIPPIE.
3. I am not clear where the subcellular location data comes from. This may be obvious to regular users of CellMap but not to me, an should be stated in the paper for other user who do not know this.

I have read this submission. I believe that I have an appropriate level of expertise to confirm that it is of an acceptable scientific standard, however I have significant reservations, as outlined above.

# Response

**Published:** 2017-11-25

**Authors:** Christian Dallago

**Affiliations:** Technical University of Munich, Germany

Dear Dr. Orchard,

Thank you for your valuable input on the tool!

With regard to point 1: we apologize for the broken database connection, unfortunately, the deployment system missed that flag and thus didn’t restart the service. We have fixed the issue and the website is now running. Up until now, I have not identified any other issues that could prevent the web server to run properly.

With regard to point 2: the software presented in this paper has a dual-purpose. On the one hand, we want to give the ability to discover protein localization and protein-protein interaction from two known sources (HIPPIE for PPI, and subcellular localization from a publication, which describes localization for the human proteome based on a consensus of experimental data and state-of-the-art prediction models ( http://doi.org/10.1038/ncomms8866)). On the other hand, we want to propose a system that can be reused on user-defined data (as long as it complies with the format the visualization tool digest, as from Figure 1) and be integrated as JavaScript visualization tool in different portals. For now, we would like to avoid having a direct integration of the portal with external tools via, for example, API calls. In an upcoming version of the portal, we will offer scripts to populate the database from different sources for the two data entities (protein localization and interaction).

PSICQUIC generates interaction data on-demand, which can later be downloaded. Obtaining the data requires some time: a user input one specific protein identifier, selects the databases to use to collect interaction data, submits a cluster job and finally gets access to the data. Searching for protein P45381 identified 80 interactions in all online databases. After several hours, the job was not finished, so we decided to lower the number of databases to fetch information from. Reducing the number of databases produced results quickly. The results page of PSICQUIC presents a table of interactions and visualizes a graph, which we could not load due to lack of compatibility with the Chrome browser. We believe it would be interesting to present CellMap at the level of this resource and will contact the authors of the tool to discuss what the best idea in this regard would be. Fetching the data from PSICQUIC as it is now and putting it into the portal requires to also normalize the PSICQUIC data and map it to protein localization data. Writing a parser for the PSI-MITAB tables is straightforward, the normalization and mapping of identifiers should occur externally to CellMap. We will create a guide on how this can be done in the next days and put it on the landing page of CellMap.

Integrating protein-molecule data and displaying these entities meaningfully is an interesting idea for the future development of the CellMap tool.

With regard to point 3: the data about protein localization stems from a publication of our group (http://doi.org/10.1038/ncomms8866). The data on protein subcellular localization for humans published through this paper was the starting point for the development of CellMap. In the current manuscript, we focused more on describing the visualization tool, rather than going into detail about how the localization data was retrieved (which in this case is by building a consensus over experimental (where available) and predicted localisations for 6 subcellular compartments). This is again because we didn’t want to develop a tool around this specific data source, but rather offer the possibility to change the origin for the localization data in the future.

We appreciate the suggestions for further data sources and data entities that can be used and integrated into CellMap. In upcoming releases, we will make sure to offer a bigger variety of data sources and scripts to populate and update the information on protein subcellular localization, and protein-protein interaction data used by the visualization tool. Additionally, we will contact the authors of PSICQUIC to discuss if it would be possible to integrate CellMap in the results page of a cluster job.

Best regards,

Christian Dallago, Tatyana Goldberg & Burkhard Rost.
